# Distractors associated with reward break through the focus of attention

**DOI:** 10.3758/s13414-016-1075-x

**Published:** 2016-03-01

**Authors:** Jaap Munneke, Artem V. Belopolsky, Jan Theeuwes

**Affiliations:** Department of Cognitive Psychology, Vrije Universiteit, Amsterdam, The Netherlands

**Keywords:** Attentional capture, Cognitive control, Attentional control

## Abstract

In the present study, we investigated the conditions in which rewarded distractors have the ability to capture attention, even when attention is directed toward the target location. Experiment 1 showed that when the probability of obtaining reward was high, all salient distractors captured attention, even when they were not associated with reward. This effect may have been caused by participants suboptimally using the 100%-valid endogenous location cue. Experiment 2 confirmed this result by showing that salient distractors did not capture attention in a block in which no reward was expected. In Experiment 3, the probability of the presence of a distractor was high, but it only signaled reward availability on a low number of trials. The results showed that those very infrequent distractors that signaled reward captured attention, whereas the distractors (both frequent and infrequent ones) not associated with reward were simply ignored. The latter experiment indicates that even when attention is directed to a location in space, stimuli associated with reward break through the focus of attention, but equally salient stimuli not associated with reward do not.

Classic models of attention have long stated that two independent control mechanisms are instrumental for attentional guidance to visual stimuli or events in our immediate surroundings. One control mechanism is assumed to be voluntary and top-down, whereas the other is assumed to be automatic and bottom-up in origin. The interaction between these mechanisms influences the way we perceive the world by selecting stimuli for further processing on the basis of our current goals (top-down) or of the stimulus’s physical saliency in the environment (bottom-up). These models of attention have been studied extensively and have long been considered the only mechanisms responsible for attentional selection (for reviews of the matter, see Corbetta & Shulman, 2002; Theeuwes, 2010; Theeuwes, Olivers, & Belopolsky, 2010).

The strict dichotomy of attentional control mechanisms was called into question when converging evidence was provided for a third category of attentional control, termed “selection history” (Awh, Belopolsky, & Theeuwes, 2012). Selection history represents the attentional bias for stimuli or stimulus features that have been selected as a target in the past. Clear examples of selection history involve a phenomenon known as *priming of pop-out* (Maljkovic & Nakayama, 1994, 1996; Theeuwes & Van der Burg, 2007), reflecting the observation that visual search is more efficient when the target-defining feature is repeated on consecutive trials, as compared to when the target feature changes. This is an important finding, as these are among the first studies to show that attention can be allocated by factors other than top-down or bottom-up control. Furthermore, selection history has been shown to underlie attentional effects, previously attributed to top-down control (Belopolsky, Schreij, & Theeuwes, 2010; Wolfe, Butcher, Lee, & Hyle, 2003; see Theeuwes, 2013, for a review).

Furthermore, the history of reward associations is a form of attentional selection based on learned associations between a stimulus and a received (monetary) reward. The rationale is that associating a stimulus with a reward boosts its representation on an attentional priority map, biasing attention toward selection of this stimulus (Awh et al., 2012). Similar to the effects of selection history, attentional guidance by reward is driven by neither bottom-up nor top-down mechanisms. Rather, it appears to reflect stimulus history: The mere association of a stimulus with a reward results in attentional capture by the rewarded stimulus, even when a reward is no longer available. The effects of reward on attention can be clearly observed in typical reward tasks (e.g., Anderson, Laurent, & Yantis, 2011) in which stimuli or stimulus features are initially associated with a reward (training phase). In a subsequent testing phase, in which rewards are no longer delivered, the crucial observation is that when the rewarded stimulus is part of the search display (but not the target) it captures attention, even when it is a nonsalient distractor (Anderson et al., 2011; Anderson & Yantis, 2012; Failing & Theeuwes, 2014; Wang, Yu, & Zhou, 2013). This phenomenon is known as *value-driven attentional capture*, and these effects are taken as evidence that stimuli that have been previously rewarded attract attention because of their learned associated value.

It should be noted that many reward studies have a training phase that is separate from the testing phase (e.g., Anderson et al., 2011; Anderson & Yantis, 2013). Typically, during training, participants repeatedly select the rewarded color (because it defines the target and the participant’s subsequent response), which then during the testing phase has to be ignored (when the previously rewarded target now appears as the distractor). As such, in this setup, selection of the rewarded stimulus in the training phase is pivotal for the outcome of the trial, as it defines the response given by the participant as well as the obtained reward. As a consequence of this setup, it has remained unclear whether and to what extent repeatedly rewarding stimuli that do not need to be selected for correct task completion can lead to value-driven attentional capture. To test whether rewarding the stimulus that defines the response, and hence whether the outcome of the trial was critical for value-driven capture, Le Pelley Pearson, Griffiths, and Beesley (2015) had participants perform a visual search task in which a shape singleton defined the target and a colored distractor singleton signaled the magnitude of the reward that could be earned on that particular trial if observers were to select the target accurately and fast enough. Importantly, the color singleton that signaled the amount of reward was never the target, and its selection was never predictive of the correct response. In fact, selecting the distractor was detrimental to obtaining a reward, due to reaction time limitations. The results of Le Pelley and colleagues showed clear value-driven attentional capture by the distractor stimulus, which cannot be attributed to an association between the rewarded stimulus and the correct response and selection criteria of the task.

These findings suggest that when valued distractors are presented in a search display together with a target, attending the valued distractor is prioritized over the target, resulting in a delayed reaction time to the target. Similar effects of reward on attentional guidance have been observed under varying experimental conditions, consistently showing that previously valued stimuli attract attention, regardless of their experimental status (e.g., whether a stimulus is a target or a distractor; Anderson & Yantis, 2013; Bucker, Belopolsky, & Theeuwes, 2015; Bucker, Silvis, Donk, & Theeuwes, 2015; Failing & Theeuwes, 2014; Wang et al., 2013).

The involuntary nature of value-driven attentional capture parallels the apparent automaticity observed in classic saliency-driven attentional capture. In his classic work, Jonides (1981) defined a number of criteria for the automaticity of attentional allocation, suggesting that capture requires a minimal attentional capacity and is resistant to suppression. Resistance to suppression reflects the mandatory character of attentional capture: Even when observers try to ignore the salient event, they are simply incapable of doing so. Although attentional capture is often described as an automatic process, Jonides showed that increasing the validity of a salient spatial cue that indicated the target location resulted in greater benefits and larger costs for valid and invalid cues, respectively. This finding suggests that observers may have some form of control over the extent to which salient events capture attention.

In line with these observations, Yantis and Jonides (1990) demonstrated that the effects of attentional capture by sudden onsets could be completely annulled by using an effective endogenous location cue. In their studies, participants had to search for and identify a target letter presented among distractor letters in a search display. The target letter could either be an onset letter among no-onset distractor letters or a no-onset target letter with one of the distractors being an onset stimulus. The results showed that reaction times to the target were not slowed by the onset of a distractor stimulus when a symbolic cue indicated the target’s location prior to its onset (Exp. 2), but only when the location cue had a high validity (Exp. 3).

Theeuwes (1991) showed similar results: Salient events outside the attentional window (evoked by an endogenous cue) did not capture attention, whereas events within an attentional window did. These findings are in line with the findings by Yantis and Jonides (1990), clearly showing that attentional capture is not completely automatic and can be influenced by the observer’s top-down attentional set. A reduced attentional window surrounding the target location has been put forward as an explanation for the observed reduced saliency-driven attentional capture (Belopolsky & Theeuwes, 2010; Belopolsky, Zwaan, Theeuwes, & Kramer, 2007; Theeuwes, 1994). Precueing the target location leads to a smaller attentional window surrounding fixation, and salient distractors falling outside of the attentional window no longer capture attention.

Due to the apparent overlap in the involuntary nature of both value-driven and saliency-driven attentional capture, the question arises whether value-associated stimuli can be ignored when observers are strongly focused on a location in space by an endogenous cue. This relation was recently investigated by Munneke, Hoppenbrouwers, and Theeuwes (2015), employing an experimental paradigm in which the location of a target letter was endogenously precued with 80% validity. The target could be presented in one of two onscreen placeholder boxes (left and right of fixation), with a distractor letter presented in the nontarget box. Critically, one of the two placeholder boxes would change to a predefined salient color with the onset of the target, its color reflecting the magnitude of the reward obtainable on that particular trial. One observation of this study showed that, when the distractor box changed to a reward color (turning the box into a valued distractor), overall slowed reaction times to the target letter were observed, with the largest increase in reaction times being for trials in which the color signaled the possibility of high reward.

The findings observed by Munneke et al. (2015) are not fully consistent with the observations by Yantis and Jonides (1990) and Theeuwes (1991), in so far as the study by Munneke et al. showed attentional capture by salient and rewarded stimuli despite endogenously focused attention at the target location. The studies by Theeuwes (1991) and Yantis and Jonides (1990) did not show attentional capture by abrupt onset distractors when a 100%-valid cue informed participants in advance of the location of the upcoming target. This discrepancy between value-driven and saliency-driven capture may indicate that value-associated stimuli occupy a preferred position on an attentional priority map, in comparison to salient stimuli. However, Munneke and colleagues’ study may not have been the most optimal way to study the influence of top-down attention on reward processing during visual search, since only two locations were used, not realistically representing the attentional constraints observed during visual search. Furthermore, an endogenous cue was used that did not predict the location of the target with 100% validity. In the present study, we aimed to investigate the relationship between attentional allocation due to reward history and top-down attentional allocation. More specifically, we addressed the question of whether value-driven attentional capture occurs under conditions in which participants can make use of an effective cue informing them of an upcoming target location. In other words, do distractors associated with reward break through the attentional focus?

In the present work, we used a design based on the study by Le Pelley and colleagues (2015), and added a 100%-valid endogenous spatial cue to the design, presented well before target onset. In this way, we were able to gauge the influence of top-down attention on value-driven attentional allocation. If valued distractors exert a stronger influence on attentional mechanisms compared to nonrewarded salient distractors, then we might expect capture to still occur, despite focused attention.

## Experiment 1

In the first experiment, we investigated whether attentional capture by valued distractors occurs when attention is fully focused on the target location by an endogenous cue (a pointer indicating the location of the target). Using an arrow to direct attention in advance to a location in space has been used in many studies as a way to endogenously manipulate spatial attention (see Jonides, 1981; Koelewijn, Bronkhorst, & Theeuwes, 2009; Theeuwes, 1991; Theeuwes & Van der Burg, 2007; Yantis & Jonides, 1990). Note, however, that some studies (e.g., Ristic & Kingstone, 2012) have shown that overlearned symbols such as arrows and possibly also pointers (as we used here) may result in orienting that is at least partly automatic. Regardless of whether orienting is purely endogenous (and/or partly automatic), using a cue before a display onset is an adequate way to manipulate the allocation of attention in the visual field.

### Method

#### Participants

We tested 12 participants (seven females, five males; mean age ± standard deviation: 25.5 ± 4.9 years) with normal or corrected-to-normal vision and no history of mental illness. All participants gave written informed consent prior to the start of the experiment. For their participation, a monetary reward was provided. The experimental procedures of this and all subsequent experiments were approved by the local ethics committee and were in accordance with the Declaration of Helsinki.

#### Stimuli and procedure

Participants were seated in a dimly lit room 75 cm from a Samsung Syncmaster 2233 monitor with a 22-in. diagonal. All of the stimuli were created and presented using Psychophysics Toolbox 3.0.12 (Brainard, 1997; Pelli, 1997) for MATLAB 2014a (MathWorks, Inc.). Eye movements were monitored using an EyeLink 1000 eyetracker (SR Research, Oakville, Ontario, Canada). A chinrest was used to assure a fixed viewing distance.

**Fig. 1:**
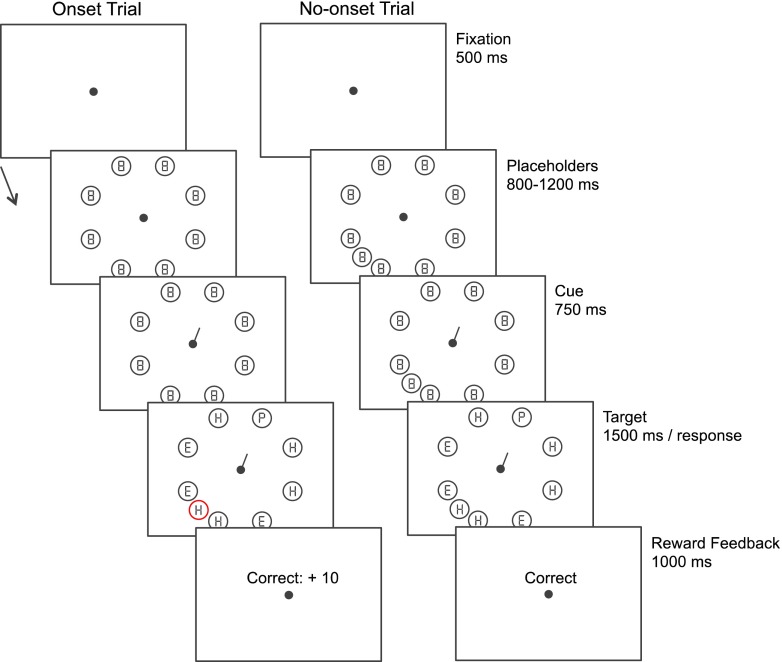
Time courses of two trial types in Experiment 1. The time course depicted on the left reflects the trials in which a valued onset distractor appeared with target presentation, and the time course depicted on the right shows the no-onset condition

The time courses of typical trials in Experiment 1 are illustrated in Fig. 1. Each trial was initiated by presenting a blank screen for 500 ms containing only a fixation dot (0.3°). Subsequently, eight circles (1.6° in diameter) surrounding fixation (radius 5.4°) would appear indicating the possible target locations. Each circle contained a figure-8 placeholder (0.8° × 0.5°) to be substituted with a letter at a later moment in the trial. Next, participants were shown an endogenous cue in the form of a small line-arrow (0.8°), which indicated the location of the upcoming target with 100% validity. After 750 ms, the placeholders turned into letters. The target letter, indicated by the cue, would turn into the letter “P” or “S,” whereas the remaining letters turned into the letters “E” or “H.” The target and distractor letters stayed on the screen until the participant had responded to the identity of the target stimulus by pressing one of two predefined keys on a standard keyboard (“z” key for S, “m” key for P) or until 1.5 s had passed. A reward screen would then appear, informing the participants about the number of points won on the trial. Prior to the start of the experiment, participants were instructed to respond as quickly as possible and not to make eye movements. In addition, they were informed that the cue was 100% valid and that the magnitude of the reward on each trial was dependent on the stimuli on the screen, their accuracy, and the speed of their responses. No further details were given with regard to the reward.

Two different trial types were used throughout the experiment. First, during an onset trial, simultaneously with revealing the target and distractor letters (by removing some of the line segments of the figure-8 premasks), a colored distractor would appear. The colored onset consisted of a circle presented at a random position between two of the original stimulus positions and never contained the target letter. On no-onset trials, an additional gray circle was present throughout the trial (see Theeuwes et al., 1999, who used a similar procedure). Similar to the other nontarget locations, this additional circle contained a placeholder stimulus that subsequently changed into a distractor letter at the moment the other placeholders changed into letters. The target location and the location of the additional stimulus in both trials types were counterbalanced over the experiment and occurred equally often at each location.

Importantly, prior work has shown that two stimuli presented close together will vie for neural representation. This competition is resolved (biased) by attention (Desimone, 1998; Desimone & Duncan, 1995). However, biased competition may entail that stimuli presented close together are processed in a qualitatively different manner than stimuli presented farther apart. Therefore, given the present design, in which the target and distractor could be presented next to each other, any influence of a closely proximate distractor on target processing does not need to reflect attentional capture. Instead, it may reflect the processes involved in resolving competition between the target and distractor. For this reason, all analyses in the Results sections for this and the following experiments have the trials removed in which target and distractor were presented directly next to each other.

Crucially, the distractor in the onset condition could be presented in three different yet equiluminant colors (red, green, and blue: 31.3 cd/m^2^). The remaining stimuli were presented in a light shade of gray (63 cd/m^2^). All stimuli were presented on a light gray background with an overall luminance of 26 cd/m^2^. These colors, which were counterbalanced over participants, indicated the magnitude of the reward that could be obtained on that particular trial with 100% certainty. Three reward levels were used: high reward (10 points), low reward (2 points), and no reward (0 points). These points translated to real money paid at the end of the experiment. However, a reward could only be obtained if the participant responded accurately and within 800 ms after target onset. If participants responded incorrectly or slower than 800 ms, no reward was administered. In the no-onset condition, participants would always obtain 0 points, as in the no-reward condition.

The experimental design consisted of six blocks of 128 trials, preceded by 30 practice trials. The four reward conditions occurred equally often and were mixed within blocks (three reward onset conditions and a no-reward, no-onset condition; 25% trials per condition). The entire experiment took approximately 80 min to complete.

### Results

#### Reaction times

**Fig. 2:**
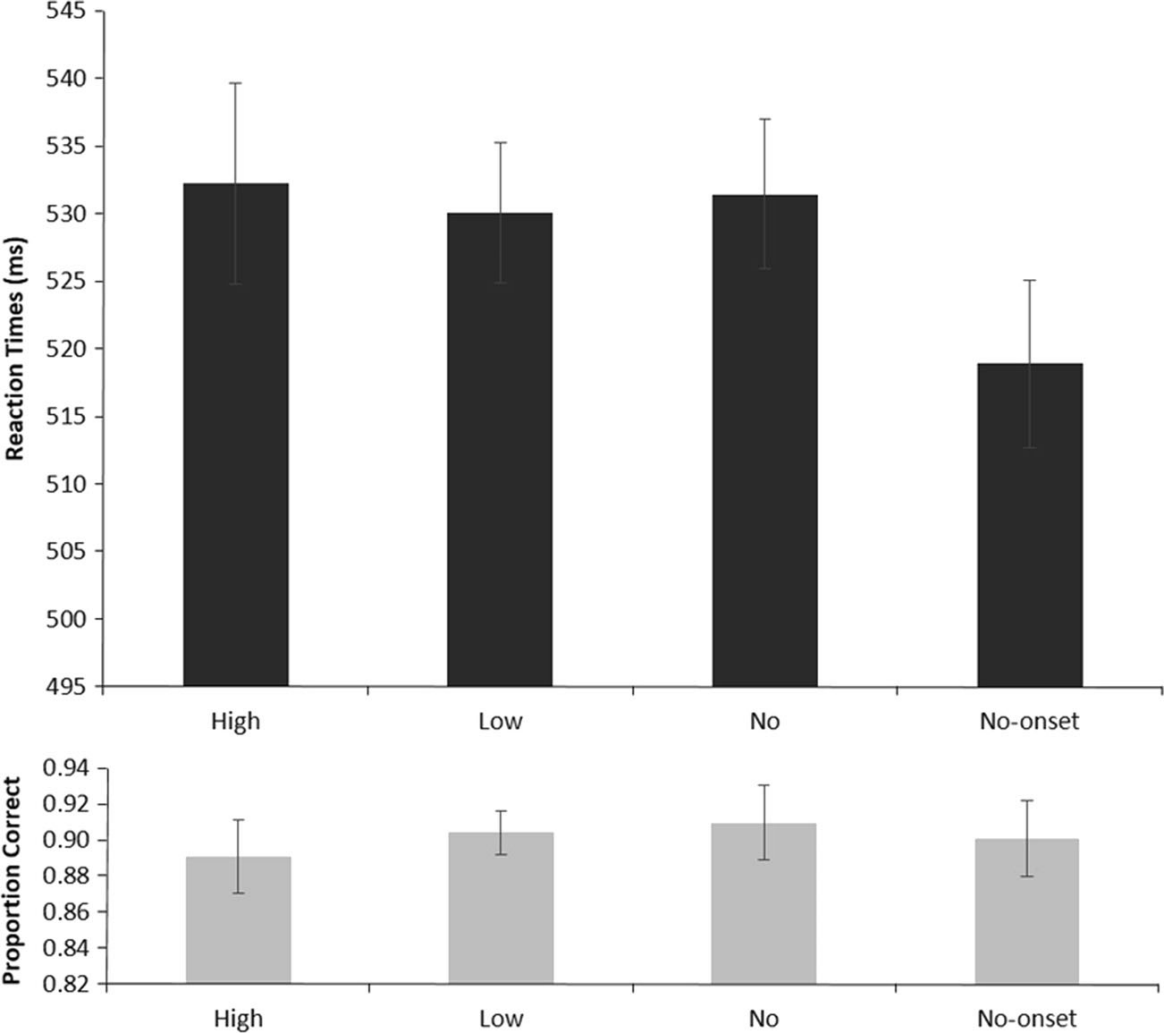
Mean reaction times (top) and accuracy scores (bottom) per reward condition. Bars represent the 95% confidence intervals for within-subjects designs (Morey, 2008)

Trials in which participants made eye movements larger than 1.25° away from fixation were discarded from the dataset (12.8%). Reaction times included in the analyses were derived from trials in which participants responded correctly (9.4% discarded) and with reaction times between 200 and 1,000 ms (fewer than 1% discarded). To gain a better understanding of how reward influences attentional allocation given full advance knowledge of the target’s location, a repeated measures analysis of variance (ANOVA) on the reaction times obtained in the experiment was performed. Figure 2 shows the mean reaction times per condition (top panel). Trial Type (high-reward, low-reward, no-reward, and no-onset) was used as a within-subjects factor. The results showed a main effect of trial type [*F*(3, 33) = 4.477, *p* = *.*01, *η*_p_^2^ = .289, power = .838], indicating that participants did not respond equally quickly in all conditions. To investigate the amounts of capture for the different conditions, we used paired-samples *t* tests to compare the reaction times obtained for the different reward levels with those obtained in the no-onset condition. The results of these planned comparisons showed that all salient distractors captured attention. At each reward level, including the no-reward condition, reaction times were significantly slower than in the no-onset condition [no onset, 519 ms; high reward, 532 ms, *t*(11) = 2.586, *p* = *.*025; low reward, 530 ms, *t*(11) = 3.107, *p* = *.*01; no reward, 531 ms, *t*(11) = 3.484, *p* = *.*005]. An additional one-way ANOVA comparing the conditions in which a rewarded distractor was presented showed no differences in reaction times between the different reward levels (*F* < 1).

#### Accuracy

A repeated measures ANOVA with Reward Level as a within-subjects factor on the accuracy data did not yield any significant differences between the reward levels (*F* < 1). Figure 2 shows the average accuracies per condition (bottom panel).

### Discussion

The results of Experiment 1 showed attentional capture for all conditions in which a salient onset distractor appeared, as compared to a no-onset condition. No differences in reaction times between any of the reward levels were observed. A straightforward explanation of these findings suggests that, despite participants being fully aware of the target location in advance, salient distractors still captured attention. However, this finding is not consistent with previous work that has repeatedly shown that salient stimuli falling outside the attentional window do not capture attention (Belopolsky & Theeuwes, 2010; Belopolsky et al., 2007; Theeuwes, 1991; Yantis & Jonides, 1990). This discrepancy may be explained by the role of reward in the present experiment. Since participants could obtain (different levels of) reward on each trial and might have preferred to monitor this information, they may not have optimally used the cue to focus attention on the target location. Reward-seeking behavior, the tendency to find and select stimuli associated with reward, may result in a less focused attention on the target location. Because attention is not fully focused on the target location, all salient stimuli, including distractors that are not associated with reward, will capture attention. In short, the presence of a possible reward evokes a reward-seeking attentional set at the cost of focused attention, which allows any salient distractor to capture attention. Note that this reasoning suggests that the presence of a possible reward-signaling stimulus leads to an attentional set that results in capture. Given that participants always knew the location of the target in advance, to further test the hypothesis that salient distractors only capture attention when they are associated with reward, we blocked the reward and no-reward conditions in Experiment 2.

## Experiment 2

The findings of Experiment 1 indicated that when obtaining a reward is possible, top-down cues are not used to their full extent. Despite our use of a 100%-valid cue, attention may not have been fully focused on the target location, leading to saliency-driven attentional capture. This would be consistent with Yantis and Jonides (1990), who showed that only when observers fully focus their attention do abrupt onsets cease to capture attention. Indeed, in their 75%-and-25% validity condition, participants adopted a more diffuse mode of attentional allocation, leading to attentional capture by abrupt onsets.

An alternative explanation is that the results we obtained in Experiment 1 might be attributed to the type of distractors we used. The reward-signaling distractors were singletons varying along two dimensions (onset and color), making them more salient than the stimuli used by Yantis and Jonides (1990). The highly salient nature of the current distractors could have resulted in attentional capture, despite focused attention at the cued target location and independent of any reward-induced top-down set. If a claim can be made that the possibility of reward leads to attentional capture, further results should show differences in reaction times between trials that lead to reward and those that do not.

In Experiment 2, the ability to obtain a reward was blocked. In this way, capture by salient distractors with and without a reward-seeking attentional set could be investigated. If the possibility of reward leads to an attenuated use of the endogenous cue, then no attentional capture would be expected in blocks without reward administration (as in Yantis & Jonides, 1990, and Theeuwes, 1991). Additionally, attentional capture by salient distractors might still occur in blocks in which reward was associated with these stimuli.

### Method

The overall methods used in Experiment 2 were highly similar to those used in Experiment 1, with a number of small changes.

#### Participants

Twelve participants were tested (nine females, three males; mean age ± standard deviation: 24.5 ± 3.3 years) after providing written informed consent. A monetary reimbursement was provided at the end of the experiment, based on time spent on the task and the reward obtained during the task.

#### Stimuli and procedure

The stimuli used in Experiment 2 were identical to those in Experiment 1. One major change was made to the procedure: The no-reward and reward conditions were tested in separate blocks. In the no-reward blocks, a salient distractor that was not associated with a reward appeared at a novel location together with the onset of the target letter (i.e., 0 points were obtained at the end of the trial). In order to keep motivation levels comparable to those in the reward blocks, participants were shown a reward feedback screen after every 16 trials. Only when participants performed with an accuracy of 80% or higher was a reward given to the participant. Crucially, the reward was not dependent on the colors presented in those 16 trials, and participants were specifically informed of this. In the reward blocks, two differently colored salient stimuli were used as distractors, reflecting either a high (10 points) or a low (2 points) reward. Reward level (high, low) was mixed within a reward block, with each level occurring equally often. As in Experiment 1, participants were informed that the magnitude of the reward in the reward blocks depended on the stimuli on the screen. However, no explicit link between a type of color and the associated reward magnitude was mentioned. In both block types (reward, no-reward), no-onset trials were mixed with the onset trials in order to provide the appropriate baseline. The no-reward part of the experiment consisted of two blocks of 128 trials, whereas the reward part of the experiment consisted of four blocks of 128 trials. A short practice block of 32 trials preceded both the reward and the no-reward blocks. In both block types, 25% of the trials consisted of no-onset trials, whereas the remaining trials contained a salient distractor. The distributions of singleton trials were 75% no-reward trials in the no-reward blocks, and 37.5% high- and 37.5% low-reward trials in the reward blocks. In a counterbalanced fashion, participants would start with either the reward blocks or the no-reward blocks. The task was the same as in Experiment 1. Again, all analyses for this experiment have the trials removed in which target and distractor were presented directly next to each other.

### Results

#### Reaction times

**Fig. 3:**
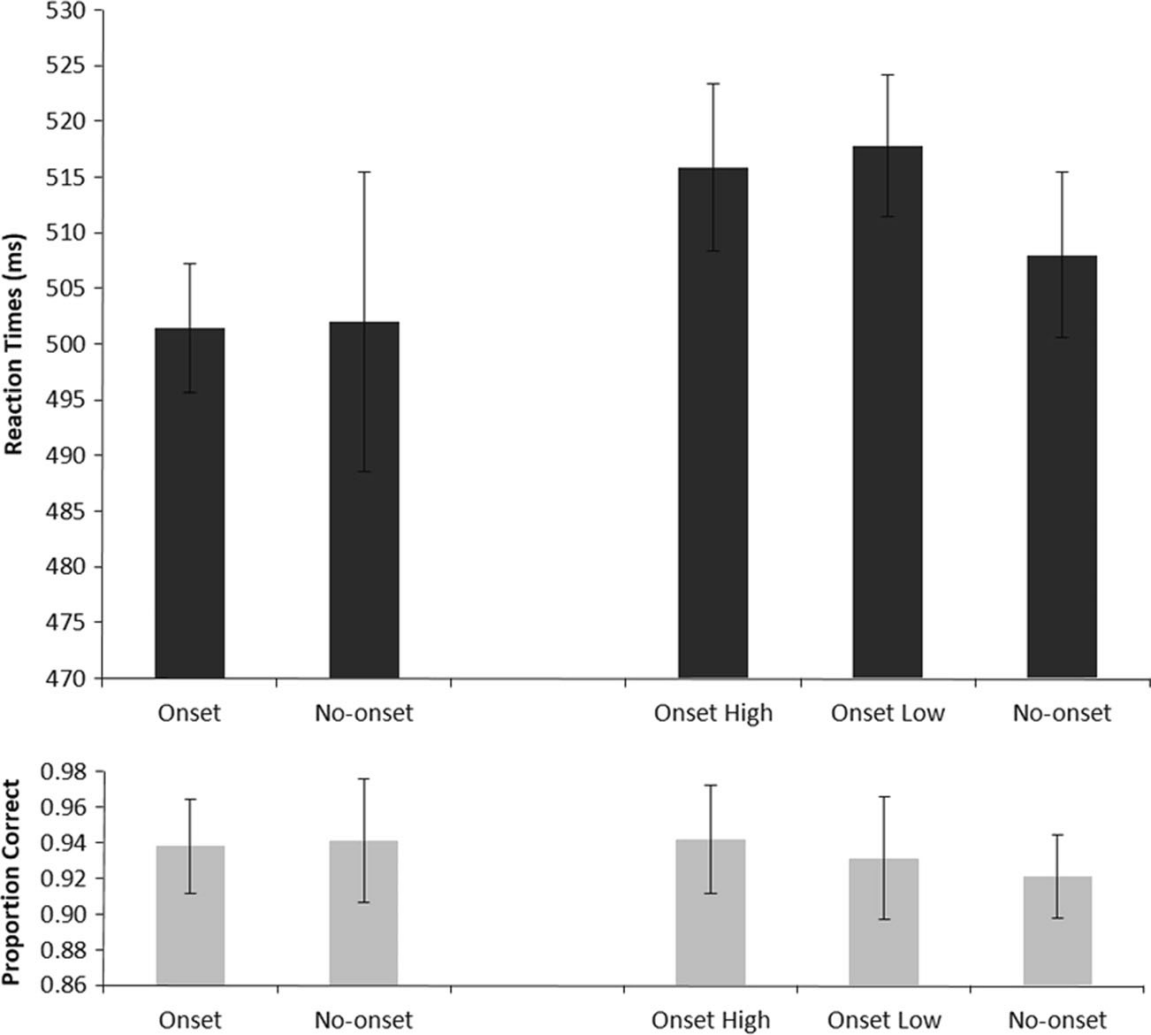
Mean reaction times (top) and accuracy scores (bottom) per reward condition. Bars represent the 95% confidence intervals for within-subjects designs (Morey, 2008). The data on the left side reflect averages observed in the no-reward blocks, whereas the data on the right side reflect averages from the reward blocks. Note that both blocks have independent measures for the no-onset trials

Trials on which the participant made eye movements more than 1.25° away from fixation were discarded from the data set (8.6%). The reaction times included in the analyses were based on trials in which the participants responded correctly (6.5% discarded) and with a reaction time between 200 and 1,000 ms (fewer than 1% discarded). To understand how the likelihood of obtaining a reward influences attentional allocation, we compared the reaction times observed in the different trial types (see Fig. 3, top panel). An initial ANOVA with only Trial Type as a factor (no-reward blocks: onset, no onset; reward blocks: low reward, high reward, no onset) showed a significant difference among all conditions [*F*(4, 44) = 3.617, *p* = *.*049, *η*_p_^2^ = .247, power = .579, Huynh–Feldt corrected]. The crucial question in Experiment 2 was to what extent salient distractors would show capture given differential reward conditions. To investigate this, planned paired-samples *t* tests were performed comparing the no-onset conditions with the onset conditions for the different block types. First, no difference was observed between the no-onset trials and the salient no-reward trials in the no-reward blocks [502 and 501 ms, respectively; *t*(11) = 0.110, *p* = *.*915], clearly showing that no attentional capture was observed within this condition.^1^ However, in the reward blocks, clear attentional capture was observed in both high- and low-reward trials. In both cases, the presence of a rewarded salient distractor significantly increased reaction times, relative to the no-onset condition [508 ms; high reward, 516 ms, *t*(11) = 2.226, *p* = *.*048; low reward, 517 ms, *t*(11) = 5.275, *p* < .001]. No difference in reaction times was observed between high- and low-reward trials (*F* < 1), suggesting that high- and low-reward-associated distractors were equally likely to capture in the present paradigm.

#### Accuracy

As in Experiment 1, no differences in accuracy were observed in a repeated measures ANOVA with Trial Type as within-subjects factor [*F*(4, 44) = 1.141, *p* = *.*350, *η*_p_^2^ = .094, power = .328]. Figure 3 shows the average accuracies per condition (bottom panel).

### Discussion

The present results clearly show that in blocks with no reward, the salient onset distractor did not capture attention. This is in line with classic findings that have shown that attentional capture is eliminated when attention is allocated at the upcoming target location (Theeuwes, 1991; Yantis & Jonides, 1990). However, small but significant attentional capture was observed in the reward blocks. These results suggest that, despite participants being informed with 100% accuracy where the target will be presented, salient stimuli still capture attention when a reward can be obtained. As in Experiment 1, no differences in reaction times between high- and low-reward trials were observed. This finding further suggests that the presence of reward might alter the observer’s strategy, such that the 100%-valid cue is not used optimally. Critically, not using the cue and engaging in a reward-seeking strategy might be detrimental to obtaining the actual reward, as a result of the search-induced increased reaction times. Thus, the presence of reward leads to altered search strategies, subsequently leading to attentional capture by salience. Any direct effects of reward on attention (i.e., value-driven attentional capture) in this experiment were obscured by these altered search strategies. In order to investigate whether value-driven attentional capture occurs despite full knowledge of the target location, a third experiment was run to force participants to use the cue, which then might discourage reward-seeking behavior.

## Experiment 3

Experiment 1 and 2 showed that the possibility of obtaining reward can lead to attentional capture by any salient stimulus, regardless of its associated value. However, in order to investigate whether stimuli associated with reward can have a more direct influence on attentional allocation (i.e., whether a rewarded salient stimulus breaks through attentional focus, whereas an equally salient but nonrewarded stimulus does not), we used a design in which the probability of the presence of a distractor was high but the distractor only signaled reward availability on a low number of trials. The low frequency of trials in which a reward could be obtained should discourage the participants from engaging in reward-seeking behavior. If value-driven attentional capture breaks through the focus of attention, then capture would only occur with the low-frequency reward-signaling distractor, but not with any other similarly salient distractor.

### Method

The same general paradigm used in Experiment 1 was used in Experiment 3, as well. A number of changes were made to discourage the reward-seeking strategy observed in Experiments 1 and 2.

#### Participants

Twenty-four participants (15 females, nine males; mean age ± standard deviation: 23.8 ± 3.4 years) took part in Experiment 3. All gave written informed consent prior to the start of the experiment. As in the earlier experiments, participants were awarded a monetary compensation at the end of the experiment based on the duration and the obtained reward during the experiment.

#### Stimuli and procedure

A number of small changes were made to the procedure of Experiment 3. First, the reward reaction time cutoff was decreased from 800 to 600 ms: Only when participants responded correctly and faster than 600 ms after target onset could a reward be obtained. This change was made so that engaging in reward-seeking behavior would be more detrimental for obtaining a reward. It further enforced the use of the endogenous cue, since its use would lead to faster reaction times. Three different distractor colors were used in the experiment, but only one of the colors led to a high reward (+10 points). The other two colors were used as no-reward colors. A high reward could be obtained only on 12.5% of the trials, which discouraged the reward-seeking strategy. One of the two no-reward colors was presented with the same low frequency (12.5%), allowing for a comparison of reward effects to effects induced by low-frequent stimuli (novelty, oddball; see Neo & Chua, 2006). The remaining no-reward color was presented with a high frequency (50% of the trials). All trial types were mixed within a block. As in Experiments 1 and 2, no-onset trials were present on 25% of the trials. Note that 87.5% of the trials were unrewarded, a design choice aimed at discouraging reward-seeking behavior. If rewarded stimuli can break through the focus of attention, then we expected the rewarded stimuli to slow down reaction times to the target, whereas the low-frequency no-reward stimulus should not have that effect. On the basis of previous studies (e.g., Yantis & Jonides, 1990) and Experiment 2, we did not expect a frequent nonrewarded distractor to capture attention. All conditions were mixed within the experimental blocks. The experiment consisted of 13 blocks of 64 trials, with the first block being a practice block that was exempt from all analyses. Trials in which the target and distractor were presented directly next to each other were not analyzed.

### Results

#### Reaction times

**Fig. 4:**
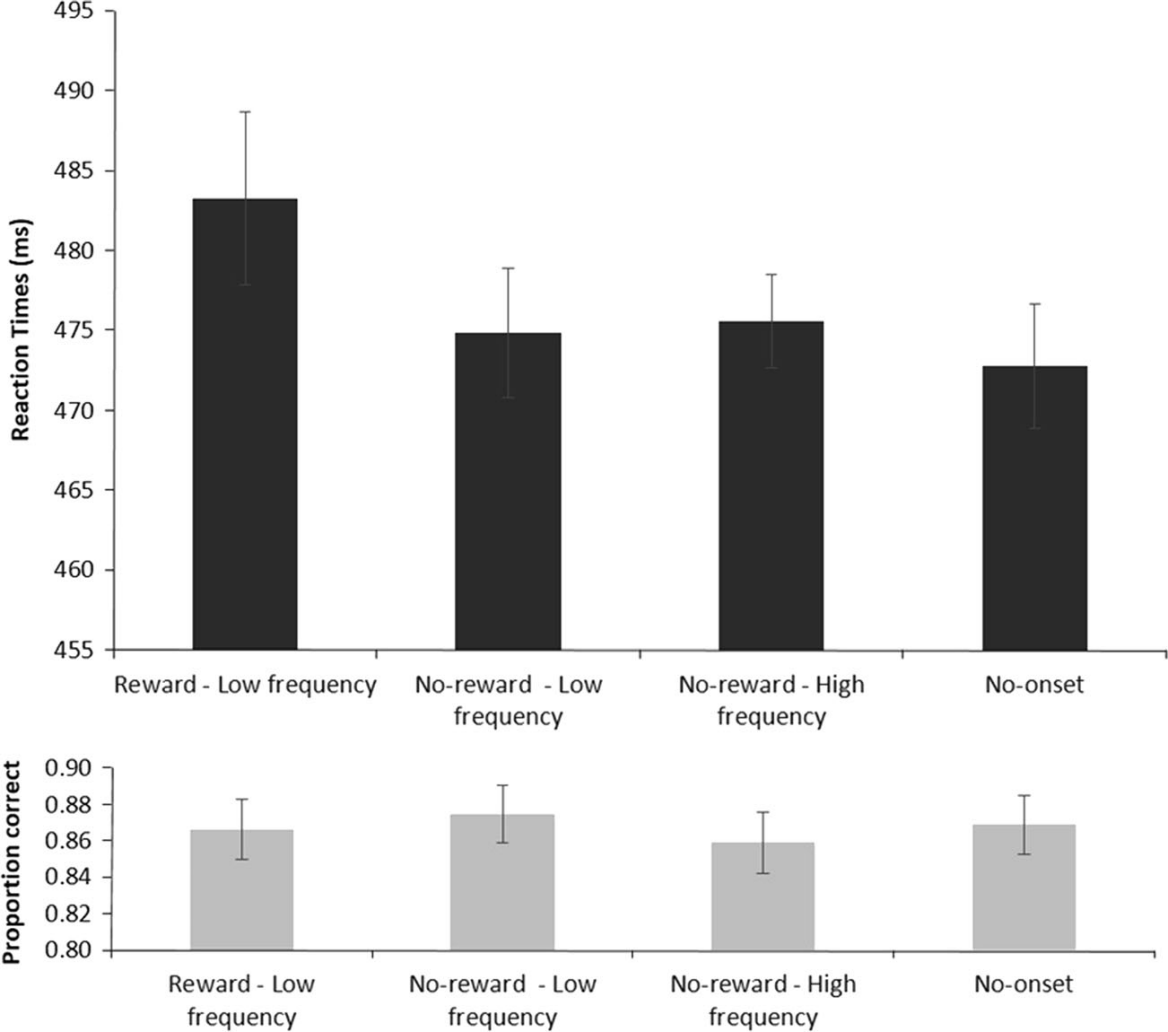
Mean reaction times (top) and accuracy scores (bottom) per reward condition. Bars represent the 95% confidence intervals for within-subjects designs (Morey, 2008)

Trials on which participants made eye movements more than 1.25° away from fixation were discarded from the data set (11.89%). The reaction times included in the analyses were derived from trials on which participants responded accurately (13.41% discarded) and with reaction times between 200 and 1,000 ms (fewer than 1% discarded). Figure 4 (top panel) shows the mean reaction times per condition. An initial ANOVA with Condition (high-reward, no-reward low-frequency, no-reward high-frequency, and no-onset) as the only factor showed that the reaction times differed over conditions [*F*(3, 69) = 4.811, *p* = *.*007, *η*_p_^2^ = .173, power = .836, Huynh–Feldt corrected]. Planned *t* tests showed that only the high-reward condition resulted in slowed reaction times to the target relative to the no-onset condition [483 and 473 ms, respectively; *t*(23) = 2.845, *p* = *.*009]. The lack of a difference between the no-onset condition and the low-frequency no-reward condition [475 ms; *t*(23) = 0.854, *p* = *.*402], as well as the nonsignificant difference between the no-onset condition and the high-frequency no-reward condition [476 ms; *t*(23) = 1.284, *p* = *.*212], indicates that participants were optimally using the cue, as no capture was observed in these conditions (Theeuwes, 1991; Yantis & Jonides, 1990).

To investigate the effects of the different distractor types on overall reaction times, a second ANOVA was run with Distractor Condition (reward, low-frequency distractor, and high-frequency distractor) as a factor. The no-onset trials were not included in this analysis. The results of this analysis showed a main effect of distractor condition [*F*(2, 46) = 4.643, *p* = *.*015, *η*_p_^2^ = .168, power = .755]. Planned paired-samples *t* tests showed that rewarded distractors yielded slower reaction times to the target than did both low-frequency distractors [*t*(23) = 2.313, *p* = *.*030] and high-frequency distractors [*t*(23) = 2.638, *p* = *.*015]. No difference in reaction times was observed between the two nonrewarded distractor colors [*t*(23) = 0.294, *p* = *.*771]. Overall, these results clearly show that rewarded stimuli capture attention, despite participants fully utilizing the endogenous cue, as reflected by the absence of capture in the nonreward conditions.

#### Accuracy

An ANOVA on the accuracy data, with Trial Type as a within-subjects factor, did not show significant differences between the different reward conditions in Experiment 3 (*F* < 1). Figure 4 (bottom panel) shows the average accuracy levels per condition.

### Discussion

The lack of attentional capture for abrupt onsets not associated with reward indicates that participants must have used the cue to optimally focus attention on the location of the upcoming target. Indeed, Yantis and Jonides (1990) demonstrated that if attention is not fully focused in advance on the upcoming target location (their Exp. 2) and if the cue is not 100% valid (but instead is 75% valid; their Exp. 3), abrupt onsets show a strong attentional capture effect. Because in our experiment abrupt onsets ceased to capture attention, we have to conclude that attention was highly focused in advance at the target location. Crucially, at the same time that participants were in the fully focused state, a distractor signaling high reward did capture attention, as reaction times for this condition were elevated relative to the no-reward onset distractor conditions. We conclude that capture by abrupt onset distractors is fundamentally different from capture by distractors that signal reward, because distractors that signal reward break through the focus of attention, whereas distractors not associated with reward cease to capture attention when endogenous attention is engaged at a location in space.

## General discussion

In the present study, we investigated whether valued distractors capture attention, given that participants were informed about the location of the upcoming target with 100% validity. In Experiment 1, we showed that despite our cueing the target location, all salient stimuli captured attention, regardless of whether or not they were associated with a reward. In Experiment 2, reward delivery was blocked. The results showed that salient distractors only captured attention when participants were engaged in a reward block, whereas capture ceased to exist when participants could not obtain a reward. In Experiment 3, participants were encouraged to utilize the endogenous cue by providing reward on only a small percentage of the trials, whereas a distractor was presented on a high percentage of all trials. Our results showed attentional capture only when a reward-associated distractor was presented, but not when an equally salient and infrequent no-reward distractor was presented.

The results of Experiment 1 appear to be at odds with those observed in a previous study by Munneke and colleagues (2015). In this study, the location of the upcoming target was cued with a validity of 80%, which is unlike the 100% validity (certainty) used in the present study. In addition, similar to the setup in the present study, in this previous study a salient outline signaled the magnitude that could be obtained on that particular trial. However, despite the lower validity of the cue, in the previous study, the salient items were not all equally likely to produce capture. The strongest effects of capture were observed for highly rewarded stimuli, and less or no capture was observed for low-rewarded stimuli. One would have expected that using a cue with a lower validity would result in participants being even more susceptible to saliency-driven capture, but this was not the case in the previous work. A number of possible reasons could explain the discrepancy between the present Experiment 1 and the results of Munneke et al. (2015).

First, Munneke et al. (2015) used a spatial cueing task with only two possible target locations. In the present study, eight possible locations were used, which significantly reduced expectations about the possible target location. A second crucial difference between the present study and the previous study is that in the present study, the target letter remained onscreen until a response was given. In the previous study, the target remained onscreen for only 200 ms. The longer presentation time for the target may have led to a different strategy, because even after participants had directed attention to the distractor, the target was still present on the screen. This strategy was not an option in the previous experiments, since the target was not present on the screen for a long enough period of time. These two factors (and the fact that the cue validity in the previous study was 80% instead of 100%) may have contributed to the attentional differences observed in in these two studies.

The results obtained in this study describe two independent ways in which reward can influence top-down attentional control settings. Reward may have both *direct* and *indirect* effects on attention. The *indirect* effect of reward on top-down attention can be observed in Experiments 1 and 2, in which we showed that the possibility of obtaining a reward leads to participants altering their search strategies, such that a 100%-valid location cue is suboptimally used in favor of reward-seeking strategies. Despite their making use of the cue, as indexed by the lack of attentional capture in the no-reward blocks of Experiment 2, attentional resources in the reward blocks remained available for processing the reward stimuli. The cue-induced attentional window was likely not small enough to merely encompass the target, but was large enough to encompass all stimuli presented onscreen, including salient distractors. This, in turn, led to classic bottom-up attentional capture by salient distractors. Both Experiments 1 and 2 showed that when reward can be obtained, all salient distractors capture attention, including no-reward distractors. However, Experiment 2 further showed that when participants were aware that they could not obtain a reward, bottom-up attentional capture was no longer present. Thus, the indirect way in which reward influences top-down attentional control settings refers to a strategic top-down setting in which residual attentional resources remain available for reward-seeking behavior. Due to this availability, classic saliency-driven, bottom-up capture occurs.

Experiment 3 showed the *direct* way in which reward can change top-down attentional settings. When the chances of obtaining a reward were relatively low (due to the low frequency of high-reward trials), participants did not engage in reward-seeking behavior, as they fully focused their attention on the cued location. Again, in this condition, the salient distractors did not capture attention anymore (see also Theeuwes, 1991; Yantis & Jonides, 1990). However, under these circumstances, while participants were in a fully focused state, reward-signaling stimuli did still capture attention. The presence of value-associated distractors was able to break through attentional top-down settings in an involuntary and automatic way.

The indirect means of value-driven attentional guidance should not be mistaken with the motivational aspects of value-driven attention. Prior work has shown that the likelihood of a reward influences task performance, due to either improved motor preparation and execution (Mir et al., 2011) or cognitive factors such as enhanced attentional processes (Sawaki, Luck, & Raymond, 2015; Small et al., 2005). However, in these and other studies of reward-induced motivation, participants were fully aware of the reward that could be obtained on each trial, through either trial-by-trial reward cueing or blocking reward distribution in the experimental design. In the present study, effects of motivation seem unlikely, since participants became slower, rather than faster, when reward could be obtained. Even in the reward blocks of Experiment 2, in which participants could obtain a reward in 75% of the trials, a slowing down of reaction times was still observed. This indicates that a mechanism qualitatively different from motivation was influencing attentional allocation. Nonoptimized use of the endogenous cue was proposed as an explanation, which in turn would alter the size of the attentional window such that attentional resources would be allocated to salient distractors, leading to bottom-up attentional capture. Therefore, it appears that the effects obtained in Experiments 1 and 2 reflect an interaction between bottom-up and top-down attentional processes.

In contrast, the *direct* influence on attention, observed in Experiment 3, was caused by the reward properties of the stimulus, much like classic bottom-up attention. The observer’s attention was involuntarily and automatically drawn to the rewarded stimuli in the visual field, despite attending a different target location. The observation that rewarded stimuli can attract attention when it is already endogenously deployed at a different target location is important, given earlier findings that salient (but unrewarded) stimuli do not capture attention under stringent top-down settings (Belopolsky & Theeuwes, 2010; Theeuwes, 1991; Yantis & Jonides, 1990). Theeuwes and colleagues (Belopolsky & Theeuwes, 2010; Belopolsky et al., 2007; Theeuwes, 1994, 2004) have argued that the lack of attentional capture by salient items is related to the size of the attentional window.

When attention is focused on a fixed location (e.g., a cued target location), the window of attentional modulation is narrow and only encompasses the location around attentional fixation. Any sudden stimulus onset falling outside the attentional window therefore will not capture attention. Nevertheless, the present experiment shows that when an unexpected rewarded and salient stimulus falls outside of the attentional window, it will still capture attention, seemingly breaking through the focus of attention. This difference in terms of capture sets value-driven attentional capture apart from saliency-driven attentional capture, such that salient items that have been associated with reward capture attention, despite prior allocation of attention to the target location, whereas salient items that are not associated with reward do not. Importantly, both distractor types were physically salient, and we cannot rule out that the observed effect was partially driven by the physical salience of the stimuli, most likely interacting with reward. What is important is that where a salient distractor could otherwise be ignored, the availability of reward ensured that this was no longer the case. A similar point can be made for the low-frequency or novel stimuli: Perhaps direct effects of reward on attention (while attention is voluntarily engaged at a possible target location) only occur when reward is combined with a stimulus that is presented rather infrequently. However, note that low frequency alone is not enough for capture to occur, as we showed in Experiment 3. This indicates that the low frequency of the distractor presentation is not what drives this effect, but instead the reward that it signals. Yet the crucial difference between the two distractor types is the association with reward that causes the observed increased reaction times, emphasizing the importance of reward associations in attentional guidance. Although a similar attentional mechanism may subserve both attentional phenomena, it is clear that value-driven attention is less influenced by a prior top-down attentional set. That is, rewarded salient stimuli appear to be strongly manifested on a hypothesized priority map, as compared to salient stimuli, leading to a quantitatively stronger influence on where attention is allocated.

Finally, because we did not employ separate training and testing phases in the present study, it can be argued that the salient distractor was not just reward-related, but also contained feedback about task performance. This feedback, rather than the reward history of the salient stimuli, may have led to attentional capture. However, in the present study, attending the distractor that signaled reward was counterproductive to the task at hand. Participants were required to search for the target and give a response. Orienting to a distractor would render this task less efficient and would decrease the chance of obtaining a reward (since attending the distractor would result is less optimal performance). Attending to the reward information was therefore not task relevant.

## Footnotes

1 Because the salient distractor in the no-reward block always had the same color, it can be argued that the lack of an effect was caused by a suppression of the distractor color, due to negative intertrial priming for trials on which the distractor was repeated (see Theeuwes & Van der Burg, 2011). However, no differences in reaction times were observed between trials on which the distractor was repeated versus not repeated (*p* = *.*48). Neither trial type (repeated and not-repeated distractor) yielded reaction times that were significantly different from those in the no-onset condition in the no-reward block (*p*s = *.*79 and *.*52, respectively).
